# A Study of Deep Learning-Based Face Recognition Models for Sibling Identification

**DOI:** 10.3390/s21155068

**Published:** 2021-07-27

**Authors:** Rita Goel, Irfan Mehmood, Hassan Ugail

**Affiliations:** Centre of Visual Computing, University of Bradford, Bradford BD7 1DP, UK; r.goel1@bradford.ac.uk (R.G.); I.Mehmood4@bradford.ac.uk (I.M.)

**Keywords:** face recognition, sibling recognition, FaceNet, VGGFace, VGG16, VGG19

## Abstract

Accurate identification of siblings through face recognition is a challenging task. This is predominantly because of the high degree of similarities among the faces of siblings. In this study, we investigate the use of state-of-the-art deep learning face recognition models to evaluate their capacity for discrimination between sibling faces using various similarity indices. The specific models examined for this purpose are FaceNet, VGGFace, VGG16, and VGG19. For each pair of images provided, the embeddings have been calculated using the chosen deep learning model. Five standard similarity measures, namely, cosine similarity, Euclidean distance, structured similarity, Manhattan distance, and Minkowski distance, are used to classify images looking for their identity on the threshold defined for each of the similarity measures. The accuracy, precision, and misclassification rate of each model are calculated using standard confusion matrices. Four different experimental datasets for full-frontal-face, eyes, nose, and forehead of sibling pairs are constructed using publicly available HQf subset of the SiblingDB database. The experimental results show that the accuracy of the chosen deep learning models to distinguish siblings based on the full-frontal-face and cropped face areas vary based on the face area compared. It is observed that VGGFace is best while comparing the full-frontal-face and eyes—the accuracy of classification being with more than 95% in this case. However, its accuracy degrades significantly when the noses are compared, while FaceNet provides the best result for classification based on the nose. Similarly, VGG16 and VGG19 are not the best models for classification using the eyes, but these models provide favorable results when foreheads are compared.

## 1. Introduction

Computer-based face recognition has always been a challenging task. It assumes that every person has a unique identity that is engraved on the face. This area has recently gained a lot of attention due to its potential use in numerous applications such as automated face matching, forensic investigations and access control. For example, the use of face recognition in forensic applications is becoming more and more common nowadays, especially when other biometric information such as fingerprints and DNA is not available. Most security organizations and civil programs, such as passport authorities and driving license departments worldwide, are opting to use face recognition to detect crime or duplicate applications. The research work in this area has progressed towards using deep learning approaches to recognize and identify the face. As a result, the accuracy of face recognition algorithms has improved significantly using deep learning. Deep learning techniques in face recognition use convolutional neural network (CNN), which are proved to be more successful as they have large quantities of training data [1]. Deep learning methods can leverage extensive datasets of faces and learn rich and compact representations of faces, allowing modern models to first perform and later to outperform the face recognition capabilities of humans.

In 2018, Wang and Deng [2] provided a helpful summary of the state of face recognition research over the last three decades, highlighting the progress of face recognition from holistic learning methods such as eigenface [3], to local feature detection, to shallow learning, and finally to deep learning methods which are currently state-of-the-art.

Although there are many effective algorithms to solve the face recognition challenges, in real-world scenarios, there are many aspects where algorithms are not foolproof to solve the face recognition problem. Challenges in real-world face recognition can be categorized into different areas, namely pose, illumination, expression, plastic surgery, aging, identical twins, and look-alike’s faces or siblings [4]. The face recognition problem for siblings is a typical one to solve and has been lately addressed by some researchers [5,6] to verify the kinship between image pairs of siblings.

Recognition of siblings is a considerable challenge for any face recognition system where two separate subjects have a very similar appearance. The other challenge face recognition systems can face when there is only part of the face is available, and it has a very similar appearance to another due to being siblings. Furthermore, the illumination, expressions, gender, age are other challenges for face recognition models while recognizing siblings. Hence it is vital to test existing face recognition models on the complex cases for sibling face recognition. If the models can perform well on complex cases, they will be able to perform exceptionally well on simple ones as well.

Thus, this work aims to explore the performance of state-of-art deep learning face recognition models for discriminating between siblings. A framework has been designed to evaluate the performance of different state-of-the-art face recognition models to discriminate between siblings’ image pairs against different covariates of Siblings’ images. Firstly, the input image pairs have been normalized. Secondly, the embeddings of these images have been extracted using state-of-the-art face recognition models. Next, the images are classified as same or different based on the similarity measures used. Finally, the accuracy of the models is evaluated based on their classification results using the confusion matrix analysis.

In this work, the performance of four different state-of-the-art face recognition models, namely FaceNet, VGGFace, VGG16, VGG19, to discriminate the siblings have been evaluated. The performance of the models is measured with respect to four covariates: Full-frontal-face pose, cropped eyes, nose, and forehead. There are many face recognitions models such as Deep face, DeepID series, Face++, etc., but the models used in this work are the best candidates as they are proved to be more accurate and have been trained on extensive data. The datasets used in this work are custom-created by cropping the face areas of the siblings to provide complex cases to models to evaluate their accuracy and robustness.

The practical aim of this work is to provide an evaluation of state-of-the-art models to select the best technique for recognizing or discriminating the siblings or look-alikes during surveillance or criminal investigations where other biometrics modalities are not available. This work has shown that distinguishing siblings is a challenging problem, and current face recognition models have difficulty discriminating between siblings when complex cases such as nose, eyes, or foreheads are provided to them.

This paper is organized into five sections. Section 2 discusses the related work on sibling face recognition or kinship verification. Section 3 details the framework prepared for this work, Section 4 outlines the datasets, evaluation matrices, experiments and describes how the performance is analyzed. Section 5 presents the discussion on the results achieved in this work. Lastly, Section 6 presents the concluding remarks.

## 2. Related Work

Biometric authentication is used in computer science as a form of identification and access control. e.g., fingerprints, palmprints, vocal and facial recognition. The initial work in this area has used approaches such as holistic learning, local feature detection, linear projection technique for feature extraction like principal component analysis (PCA) or linear discriminant analysis (LDA), etc.

In 2010 Leng et al. [7] proposed a novel approach for face and palmprint recognition in the Discrete Cosine Transform (DCT) domain by dynamic weighted discrimination power analysis to modify the discrimination ability of the selected DCT coefficients. They have used three processes to enhance recognition. In the first process, the useless coefficients have been discarded. As a second process, useful coefficients have been selected, and lastly, they have enhanced the discrimination ability by dynamic weight adjustments. Their experimental results have shown that their methods have outperformed the existing methods in the DCT domain.

In 2011, Leng et al. [8] again proposed a novel method for feature extraction in biometrics over the random projection (RP) using two-directional and two-dimensional approaches. This approach directly projects the image from high dimensional space to low dimensional space to extract the vectors in two directions. Their method had shown its effectiveness when experimental results were compared to existing techniques.

The work in biometrics has advanced towards the use of deep learning methods for face recognition. A human face contains a lot of identity information about the individual. Researchers have proposed various methods in differentiating similar-looking faces or siblings. However, the literature around the siblings or kin is relatively scattered and is quite inconsistent. Many novel ideas have been developed to solve the kinship verification problem, e.g., [9,10]. The use of similarity measures is also helpful to find whether two images are similar or different or to assess image quality. Wei et al. [11] have used an adverse similarity metric learning for kinship verification among ambiguous test pairs such as significant age gaps or gender differences between siblings. Their method contains two faces, namely, confusion and discrimination. In the confusion phase, they generated the ambiguous image pairs from original pairs to confuse the similarity metric. A robust similarity metric is formed by continuous discrimination between original and generated pairs in the discrimination phase. Their experimental results have shown the competitive accuracy than the state-of-the-art methodologies.

Social science researchers have investigated the ability to differentiate siblings from face images. Wang et al. [12] have presented a novel heterogeneous similarity learning method to provide a more practical kinship verification based on kinship relations having their characteristics acquired from parents and some common genetic attributes. They have learned a similarity function that captured the familiarity among kinship relations and the geometry of each relation. Then their method was determined by the fusion of the similarity models from multiple feature representations. The reciprocal information in multi-view kin data can be used to obtain refined information. Their experiments on large-scale KinFaceW datasets have shown that the accuracy of their methods is superior to state-of-the-art algorithms in terms of verification, and it is more practical for kinship verification in real-world conditions.

Lu et al. [13] proposed a novel and efficient similarity index for face recognition based on structured similarity, feature similarity to find the difference between images. They have tested its performance on three large datasets under conditions of Gaussian noise. Their simulation results had outperformed the well-known structured similarity index measure (SSIM) and feature similarity index measure (FSIM) approaches in their efficiency of similarity detection and recognition of human faces.

Qin et al. [14] have proposed a novel metric learning approach to learn a similarity measure between pairs of images derived based on statistical inference perspective to indicate the traits of a similarity proportion that captures the resemblance between identities. They have improved the performance of kinship verification by using their method to execute the fusion of estimators further propose an ensemble nonlinear multi-metric learning (ENMML) method to perform multiple estimators’ fusion to improve the performance of kinship verification. They have experimented with their approach on three publicly available kinship datasets, namely, KinFaceW-II, Cornell KinFace, and UBKinFace datasets, with competitive performance.

Rakshit et al. [15] have proposed a face identification system for visible, look-alike, and post-surgery face images of individuals. They have used some novel variants which are exploited from local graph structure (LGS), which attempt to improve the performance of the face identification system under various conditions such as pose changes, facial expression changes, illumination, makeup, accessories (glasses), and facial complexity (look-alikes and plastic surgery). They have represented each pixel and its neighbor pixel to generate the LGS, which is then used to generate a binary pattern for each pixel. This binary pattern is then used to generate a concatenated histogram. This histogram has then used state-of-the-art classifiers to match and identify the images. Their experimental results have led to a concrete identification system invariant to different challenges since the images are not needed to be pre-processed as each variant acts with local face structure while other effects are not used.

Wang et al. [16] have done a comprehensive survey in 2020 to systematically categorize and evaluate the methods that emerged for kinship verification over the last decade. Then they have focused on the overall difficulties and challenges in the practical applications from the perspective of pattern recognition and computer vision. They have reviewed the performance of state-of-the-art face recognition methods and some novel algorithms on popular kin databases. Their survey has summarized that though many efforts are made in kinship verification, it will need improvements due to the complexity of this problem and its wide practical applications.

Lopez et al. [17] have compared the performances of humans and machines in kinship verification. They have investigated the state-of-the-art methods in automated kinship verification in both images and videos and compared their performance with the human interpretation of the verification of the same images. Their result shows that automated kinship verification is more accurate than human kinship verification in images and videos.

Shadrikov [18] has proposed a novel standard for automated kinship recognition. His approach is based on RetinaFace and ArcFace for face registration and face verification, respectively. He has used his Wild Data Challenges approach by designing a pipeline for fine-tuned face verification models for visual kinship recognition. His experimental results show that the approach has given the best performance in recognizing families in Wild Data Challenge.

The dataset used in this work has been created by Vieira [19] et al. by constructing four datasets with high-quality images shot in controlled conditions to detect siblings in image pairs. They first figured out how the different attributes contribute to the sibling classification and comparing automatic and human classification capabilities, and finally, they generalized the properties of the classifier. The results of their work by combining features of different nature have achieved significantly higher accuracies than the human classification accuracies.

Dornaika et al. [20] proposed a novel framework that leverages deep facial features for kinship verification by incorporating three fusion levels. Their fusion levels first select the most relevant features, then it exploits a kinship-based multi-view metric learning method and finally merges classifiers’ responses. They have used pre-trained VGG-F and VGGFace models to select the features. They conducted the experiments on KinFaceW-I and KinFaceW-II, and their results show that their framework outperforms state-of-the-art techniques without the use of data augmentation tailored for the kinship verification problem.

A couple of the similarity measures used in this work is based on the research conducted by Vo and Lee in 2017 [21] for face recognition using Euclidean distance and Minkowski distance, where they have improved the accuracy of collaborative representation-based classification by minimizing the Euclidean distance between face images and used a two-stage classifier for training faces. Their results with challenging datasets show that their approach had outperformed the state-of-the-art models.

Zahir et al. [22] have looked over human behavior using facial expression analysis. They have used some famous TV series videos in order to predict human behavior from their facial expressions. They have proposed a lightweight convolutional neural network in order to recognize facial expressions. They have achieved it in four steps. In the first step, they have detected and tracked the faces utilizing the Viola-Jones algorithm and Kanade-Lucas-Tomasi algorithm. As a second step, they classified the faces using HOG features and SVM classifier. Next, they recognized the facial expressions using their proposed method, and lastly, they have predicted the human behaviors based on their facial recognition and expression occurrences. They have performed a subjective evaluation to analyze the performance of their method, and their results were convincing for the aimed problem.

Recently, Elmahmudi and Ugail [23] have proposed a research on face recognition using partial or imperfect facial data by extracting the features from an imperfect face and matching those features with a database of full-frontal faces using a state-of-the-art convolutional neural network-based architecture with a pre-trained VGGFace model to extract the features. Their results show that individual parts of the face have low recognition rates. However, the recognition rate is high when these parts of the face are combined and presented as probes.

The latest work on kinship verification has been done by Yan and Song [24], where they have proposed a deep relational network that exploits multi-scale information of facial images for kinship verification from the local regions. They have used two CNNs that share parameter to extract different scales of features. Their results have shown good performance in identifying kinship from a pair of images.

It has been observed that there are a lot of studies available for sibling or kinship verification using face recognition and deep learning methods using various state-of-the-art face recognition models. Still, they are pretty sparse and not conclusive. This study aims to fill this void by evaluating various state-of-the-art face recognition models’ performance to distinguish between siblings from the input image pair of their full-frontal faces, cropped eyes, nose, or foreheads. In this work, four pre-trained models, namely FaceNet, VGGFace, VGG16, and VGG19, are used to extract the embedding of the input image pairs and then, five different similarity measures, namely cosine similarity, Euclidean distance, SSIM, Manhattan distance, and Minkowski distance are used to evaluate the accuracy, precision, and misclassification rates of each model on the different face area of the input image pairs of siblings.

## 3. Framework

**Figure 1:**
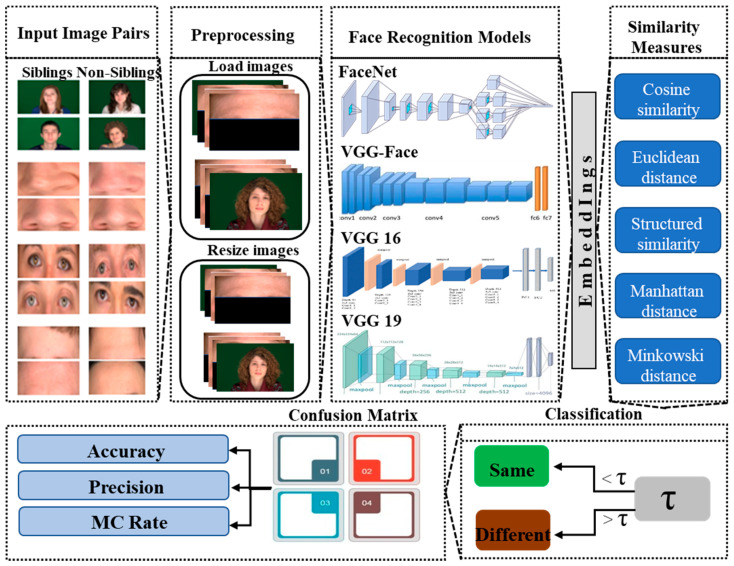
An overview of the proposed facial appearance-based sibling identification framework explores the potential and limitations of state-of-the-art face recognition models.

Face recognition is more challenging when comparing the images of similar-looking faces or siblings. This differentiation can be challenging in two ways. In some scenarios, two persons who are not siblings, but their facial appearance is quite similar. Thus, a face recognition model can classify them as the same person. On the other hand, face recognition models can also classify the actual siblings as different persons when their facial appearance is not similar. This work explores the accuracy of state-of-the-art face recognition models in some challenging scenarios, as explained above when used to differentiate between siblings. The overview of the framework used in this work is shown in Figure 1. The framework is used on image pairs of the full-frontal-face pose, cropped eyes, nose, and foreheads of the siblings and non-siblings, which are pre-processed and passed to the state-of-the-art models to differentiate between them. In the following subsections, the models and similarity measures used will be described.

### 3.1. Face Recognition Models

There are many state-of-the-art face recognition models for image comparison. In this work, the accuracy of four pre-trained state-of-the-art models, namely, FaceNet, VGGFace, VGG16, and VGG19, are evaluated when they are used to distinguish between siblings. In 2015, the researcher at Google had achieved the best results on various popular face recognition datasets and that system called FaceNet [25]. The FaceNet system is a third-party open-source implementation of the model and available as pre-trained models. The FaceNet system is useful to extract face embeddings that are high-quality features from faces that can train a face recognition system.

It learns a mapping from face images to a compact Euclidean space where distances directly correspond to a measure of face similarity [13], i.e., faces of the same person have small distances and faces of different people have large distances. FaceNet uses a deep convolutional network trained to precisely optimize the embedding itself, instead of intermediate bottleneck layers as in previous deep learning approaches. It is a robust and efficient face recognition system, and the general nature of the extracted face embeddings provides the approach to a range of applications.

FaceNet is a face recognition system described by Schroff et al. at Google in 2015 [25]. This model is a deep convolutional neural network trained via a triplet loss function that reassures vectors for the same identity to be a smaller distance. On the other hand, vectors for different identities are on the more considerable distance. This system extracts high-quality features from the given images and predicts a 128-element vector representation of these features. This vector representation is called embeddings. The critical innovation in this work was to create the image embeddings directly instead of extracting them from an intermediate layer of the model. FaceNet is trained on an extensive dataset and is quite good and widely used in face recognition. Thus, the accuracy of this model has been evaluated in this work.

Another most popular and widely used model in face recognition is VGGFace [26] which refers to a series of models developed for face recognition by members of the Visual Geometry Group (VGG) at the University of Oxford. VGGFace model is used for face verification by calculating the embeddings of the dace image and comparing it with the embeddings calculated for another face by calculating the similarity measures such as cosine distance or Euclidean distance and determining the face match by comparing the distance to a predefined threshold [27]. It consists of 11 1ayers in which eight convolutional layers and three fully connected layers. The model was trained with a vast dataset containing 2.6 M face images of the same number of individuals. The architecture of VGGFace contains 38 layers, and the input image size for this model is 224 × 224. VGGFace is relatively easy to train and very popular in visual computing for face recognition. This work aims to evaluate its accuracy for face recognition in challenging scenarios like discriminating between siblings or similar-looking faces.

Another CNN model is VGG16 which attains ~92% accuracy on ImageNet experiments, which is a dataset of over 14 million images belonging to 1000 classes. This model was presented by Simonyan and Zisserman from the University of Oxford [28] and was one of the famous models submitted to ILSVRC-2014 [29]. This model replaces the large kernel-sized filters of AlexNet [30] with multiple small-size (3 × 3) kernels to improve the accuracy. Like VGGFace, the input to conv1 layer in VGG16 is fixed to 224 × 224 RGB image. It consists of 16 convolutional layers and is very appealing because of its uniform architecture. VGG16 was trained on Nvidia Titan GPU for 2–3 weeks; hence it is prolonged to train and provides very high accuracy, but it needs high-end system configuration and increased model size.

VGG16 is a state-of-the-art model which is trained on various classes of objects and outperformed the previous generation of models in the ILSVRC-2012 [31] and ILSVRC-2013 [32]. Hence it is chosen to evaluate its accuracy in different scenarios like cropped areas of the faces.

VGG19 is a variant of the VGG model [28], which in brief consists of 19 layers (16 convolution layers, three fully connected layers, five MaxPool layers, and one SoftMax layer). This pre-trained network can classify images into 1000 object categories, such as keyboard, mouse, pencil, and many animals. As a result, the network has learned rich feature representations for a wide range of images. The network has an image input size of 224-by-224. VGG19 has increased depth with tiny (3 × 3) convolution filters, which showed a significant improvement in prior-art configurations by pushing the depth to 19 weight layers. Like its predecessor, VGG16, this model is also prolonged to train and provides very high accuracy but at the cost of increased model size and needs high-end configurations. VGG19 is chosen for these experiments as it is supposed to be high in accuracy when classifying the partial faces.

### 3.2. Full and Partial Face-Based Face Recognition

In most real-world scenarios, it is easy to recognize faces as the input is a full-face image but, in some scenarios, like in surveillance or criminal investigations where a person needs to be tracked, and only a part of the face like eyes, nose, etc. is available as input. This work analyzes the above-stated four models based on this data whether they can recognize and classify the person as a sibling.

For the given input dataset *M* containing image pairs of siblings full-frontal-face, cropped eyes, nose, or forehead, a threshold *τ* [33] has been defined for each of the above-stated models concerning each area of the face. Now images in each pair *i*, where *i* is the number of image pairs, are converted to an array using the TensorFlow pre-processing library and resized to the shape required by the respective model as stated above. For example, images are resized in 224 × 224 for VGGFace. The embeddings of the pre-processed images are then extracted using the respective model to compare them using the similarity measures. Similar work is also carried on the non-siblings’ images to find the model’s accuracy, precision, or misclassification rate. Algorithm 1 shows the whole process of extracting the embeddings and similarities for image pairs of siblings or non-siblings.

**Algorithm 1** Evaluating models to differentiate siblings.
***Input:***
*Image Pairs of Siblings M with m pairs*

*τ = threshold array defined for each similarity measure*

*mod = respective model*

***for***
*i = 1 to m **do***
      ***for** each image in pair **do***         *I_1_ → read image;*         *I_1_ → resize image;*         *I_1_ → pre-process image;*         *P_1_ = extractEmbeddings(mod(I_1_));*         *I_2_ → read image;*         *I_2_ → resize image;*         *I_2_ → pre-process image;*         *P_2_ = extractEmbeddings(mod(I_2_))*      ***end***
***end***

*cosine*
*→*
*cosine_distance(P_1_, P_2_)*

*euc → euclidean_distance(*
*P_1_, P_2_*
*)*

*ssim → structured_similarity(*
*P_1_, P_2_*
*)*

*manh*
*→*
*manhattan_distance(*
*P_1_, P_2_*
*)*

*mink → minkowski_Distance(*
*P_1_, P_2_*
*)*

***for***
*j = 1 to τ.length **do***
      *if distance < τ[j]*         *distance_result → “Same”*      *else*         *distance_result → “Different”*
***end***


### 3.3. Evaluation Matrices for Face Recognition Model

When the above stated four models are analyzed for their efficiency on sibling datasets, the accuracy, precision, and misclassification rate [34] are calculated for each model on each dataset used, i.e., full-frontal-face, cropped eyes, nose, or forehead to evaluate the classification rate efficiency of state-of-the-art models by making it a two-class problem using the non-sibling datasets used in the experiments and forming the confusion matrix for each model with each dataset used. These advanced evaluation matrices have been used to evaluate the efficiency of the face recognition model as classification accuracy calculated by correct predictions/total predictions hides the necessary details to better understand the performance of the face recognition model.

#### 3.3.1. Accuracy, Precision, and Misclassification Rate

A confusion matrix [35] summarizes prediction results on a classification algorithm or face recognition model when used to classify. It gives insight not only into the errors being made by the classifier but, more importantly, the types of errors that are being made. The event row is assigned as “positive”, and non-event row is assigned as negative, and the predictions are assigned as “true” or “false”.

In this work, experiments are conducted on the image pairs of siblings and non-siblings, which are always different. Hence the true cases are evaluated on the sibling dataset, and false cases are evaluated on the non-sibling dataset:

- True positive (*TP*): These cases are identified when the image pair of siblings are compared with actual cases as different and predicted cases are also different, i.e., models have classified them as different persons.
- True negative (*TN*): Identified on comparing sibling’s image pairs and models have identified them as the same person but different.
- False positive (*FP*): These are the cases when image pairs of non-siblings are compared, which are different, and models have also identified them as different persons.
- False negative (*FN*): When non-siblings are compared and models have classified them as the same persons, the case is identified as a false negative.

Equations (1)–(3) depict the calculation of accuracy, precision, and misclassification rate calculated based on the cases identified, respectively:

(1) $$\mathrm{Accuracy}=\frac{TP+TN}{TC}$$

with TC is total cases:

(2) $$\mathrm{Precision}=\frac{TP}{Predicted\;Yes}\;$$

(3) $$\mathrm{Misclassification}\;\mathrm{Rate}=\frac{FP+FN}{TC}$$

#### 3.3.2. Similarity Measures

Five standard similarity metrics namely, cosine similarity, Euclidean distance, structured similarity, Manhattan distance, and Minkowski distance [13] have been employed to categorize pairs of images of either siblings or non-siblings. These similarity measures are robust in comparing the image vectors in machine learning and face recognition models. A threshold is defined for each similarity measure, and the respective similarity is matched against this threshold to classify the image pair as same or different. Cosine similarity is a measure of similarity between two non-zero vectors of inner product space and can be calculated as:

(4) $$\mathrm{Similarity}=\cos\left(\theta \right)=\frac{A\cdot B}{\left|\left|A\right|\right|\;\left|\left|B\right|\right|}=\frac{\sum_{i=1}^{n}A_{i}\;B_{i}}{\sqrt{\sum_{i=1}^{n}Ai^{2\;}}\;\sqrt{\sum_{i=1}^{n}Bi^{2\;}}}$$

where $A_{i}$ and $B_{i}$ are components of vectors *A* and *B*, respectively.

The Euclidean distance [13] between two vectors *a* and *b* in the Euclidean space is the length of a line segment between *a* and *b* and can be calculated as,

(5) $$Euc\left(a,b\right)=\sqrt{{\left(b_{1\;}-a_{1}\right)}^{2}+{\left(b_{2\;}-a_{2}\right)}^{2}\;}$$

On the other hand, Structured similarity [36] is used for measuring the similarity between two images and calculated between two images *a* and *b* of standard size *N* × *N* is,

(6) $$SSIM\left(a,b\right)=\frac{\left(2\mu_{a}\mu_{b}+s_{1}\right)\;\left(2\sigma_{ab}+s_{2}\right)}{\left(\mu_{a}{}^{2}+\mu_{b}{}^{2}+s_{1}\right)\;\left(\sigma_{a}{}^{2}+\sigma_{b}{}^{2}+s_{2}\right)}$$

with: $\mu_{a}$ the average of *a*, $\mu_{b}$ the average of *b*, $\sigma_{a}{}^{2}$ the variance of *a*, $\sigma_{b}{}^{2}$ the variance of *b*, $\sigma_{ab}$ the covariance of *a* and *b*, $s_{1}={\left(j_{1}K\right)}^{2}$, $s_{2}={\left(j_{2}K\right)}^{2}$ two variables to stabilize the division with a weak denominator, *K* the dynamic range of the pixel value, $j_{1}=0.01$ and $j_{2}=0.03$ by default.

Manhattan distance [27] is the distance between two vectors measured along axes at right angles. In a plane with $p_{1}$ at ($a_{1}$, $b_{1}$) and $p_{2}$ at ($a_{2}$, $b_{2}$), it can be calculated as:

(7) $$\mathrm{Manhattan}\;\mathrm{distance}\;\left(D\right)=\left(\left|a_{1}-a_{2}\right|+\left|b_{1}-b_{2}\right|\right)\;$$

The Minkowski distance [37] is a metric in a normal vector space that can be considered a generalization of both the Euclidean distance and Manhattan distance. The Minkowski distance of order *p* (where *p* is an integer) between two points *P* and *Q* is defined as,

(8) $$\mathrm{Minkowski}\;\mathrm{Distance}\;\left(D\right)={\left(\overset{n}{\underset{i=1\;}{\sum }}|P_{i}-Q_{i}{|}^{p}\right)}^{\frac{1}{p}}$$

The similarity between the image pairs of siblings and non-siblings is calculated as stated above. Then those images are classified as of the same person or different persons based on the threshold taken for each similarity measure for each face recognition model and each dataset used. Once the images are classified, they are passed to the confusion matrix as one of the events or non-event cases (TP, TN, FP, or FN) to evaluate the accuracy, precision, and misclassification rate of the respective face recognition model.

## 4. Experiments and Results

A comprehensive set of experiments have been conducted in this study on sibling datasets using four state-of-the-art face recognition models, namely, FaceNet, VGGFace, VGG16, and VGG19. These experiments are carried out on different parts of the faces of siblings. The frontal face images from the popular SiblingsDB [19] dataset have been used to achieve this work. This dataset has a variety of high-quality images of siblings and available on request to the research community. A subset of sibling image pairs has been created for the full-frontal pose, and they have been cropped to create separate datasets for eyes, nose, and forehead images pairs of siblings.

Similarly, another dataset for non-siblings has been created using the random images from SiblingsDB [19]. Nose, eyes, and forehead are cropped from full face images to create parts of face datasets of image pairs for non-siblings. The experiments have been carried out on the image pairs of full-frontal face, and parts of faces: nose, eyes, and forehead of siblings and non-siblings. In each case, numerous similarity measures have been used on each state-of-the-art face model to evaluate its accuracy. In this following subsection, the datasets used, experiments carried out on different datasets, and their results will be detailed.

### 4.1. Datasets

The SiblingsDB constructed by Vieira et al. [19] is used to conduct these experiments, which contains different datasets depicting images of individuals related by sibling relationships. The images in this dataset are organized in two different DBs, namely, HQfaces, and LQfaces. There are 92 high-quality pairs of 184 individuals in HQfaces, while on the other hand, LQfaces contains 98 pairs of siblings from the internet.

The subjects in images in HQfaces are voluntary students and employees of Politecnico di Torino and their siblings. These images are shot in a controlled environment with a constant background by a professional photographer in a high resolution of 4356 × 2832.

These images are further organized into three discreet datasets, namely HQf, HQfp, and HQfps, containing expressionless frontal images on 92 siblings pairs, 79 sibling pairs of frontal and profile images each, and 56 image pairs, respectively. Each individual is represented by four images (two expressionless frontal and profile and two smiling frontal and profile images).

**Figure 2:**
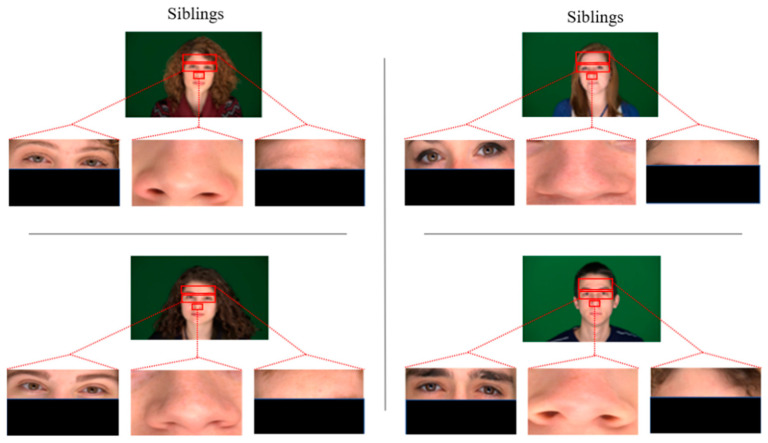
Images from the datasets used: two pairs from siblings’ full-frontal pose, cropped nose, eyes, and foreheads.

**Figure 3:**
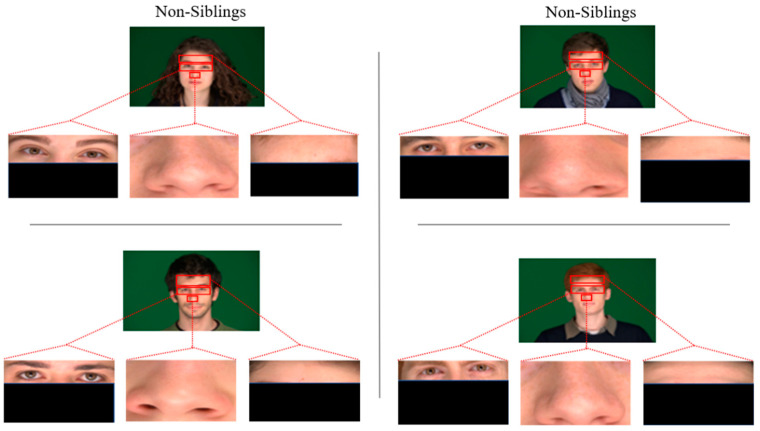
Images from the datasets used: two pairs from non-siblings’ full-frontal pose, cropped nose, eyes, and foreheads.

To evaluate the accuracy of state-of-the-art face recognition models, two subsets of 60 images each (30 image pairs) of siblings for positive pairs and randomly chosen non-siblings for opposing pairs are formed. The individuals in the datasets are 65% male and Caucasian. These datasets have been diversified further by cropping to create three more datasets of their cropped eyes, nose, and foreheads for each pair of images of siblings and non-siblings. Figure 2 and Figure 3 show some sample images of sibling image pairs and non-sibling, respectively.

Since the datasets used in these experiments do not distinguish between training and testing sets, the accuracy, precision, and misclassification rate of the models used are computed by calculating the similarity between image pairs. The image pairs of positive cases and negative cases have been represented as vectors, and by comparing these vector representations, it is decided whether the images compared are classified as of the same person or different persons. The comparison between images is made using five popular similarity measures, namely, cosine distance [13], L2 Norm or Euclidean Distance [13], structured similarity (SSIM) [36], Manhattan distance [27], and Minkowski distance [37]. To differentiate the images, a threshold is used for each similarity measure so that images can be classified as same or different, comparing the similarity metric score with the threshold. Once the similarity between positive and negative image pairs has been calculated, a confusion matrix has been generated for each model on each similarity measure to evaluate the model’s accuracy, precision, and misclassification rate on the dataset used in the respective experiment.

### 4.2. Experiments on Full Frontal Face

**Figure 4:**
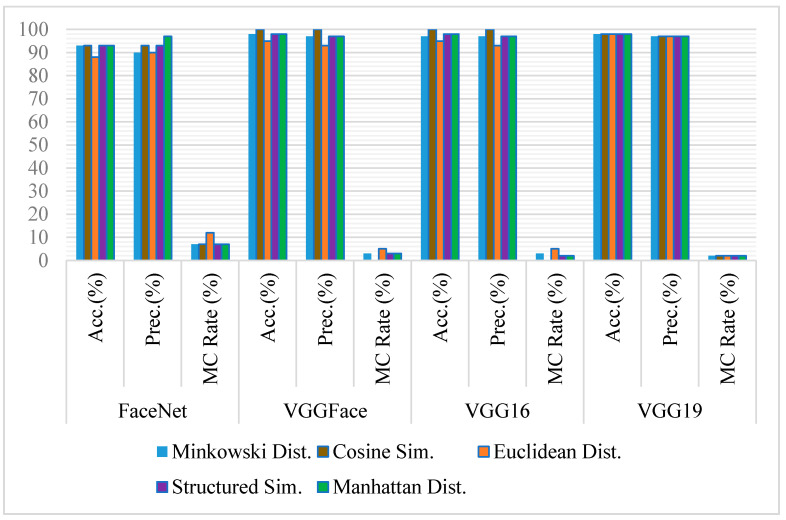
Comparative analysis of FaceNet, VGGFace, VGG16, and VGG19 for face recognition on full-frontal-face image pairs using Accuracy, Precision, and Misclassification rate.

The accuracy of the state-of-the-art models has been computed on full-frontal face positive and negative image pairs datasets with each similarity measure separately. Some sample images of positive and negative pairs are shown in Figure 2 and Figure 3. The images have been represented as vectors with each model used, and the similarity between those representations has been calculated based on the threshold taken. Figure 4 summarizes the accuracy, precision rate of each model on the full-frontal face. It has been observed that all the models have at least around 90% accuracy when the full-frontal face is compared of siblings for true cases and non-sibling faces for false cases. The accuracy of the FaceNet model is constant on all similarity measures except Euclidean distance, where it has a bit degraded where facial hair or facial marks are involved. It is also observed that these persons and similar-looking faces and hair cut have caused FaceNet to classify them as the same person. FaceNet has also classified two out of 30 such images as the same person with other similarity measures.

**Figure 5:**
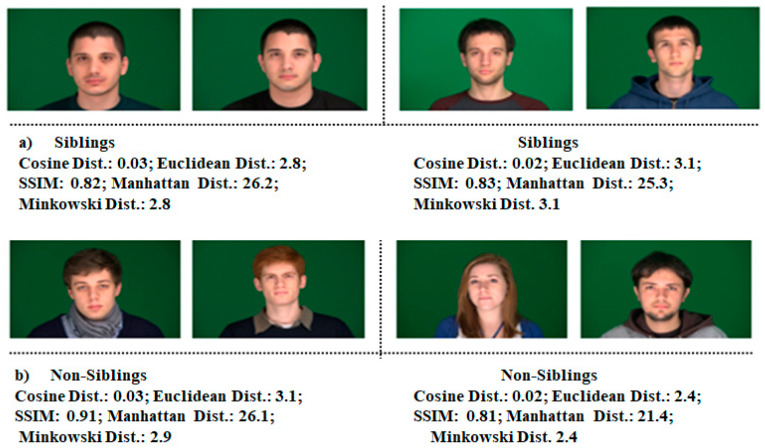
(**a**) Images of the dataset used: two pairs from Siblings full-frontal pose where all four models have classified them as same person. (**b**) Images of the dataset used: two pairs from non-Siblings full-frontal pose where all four models have classified them as same person.

On the other hand, both VGGFace and VGG16 have yielded almost similar results where they have been 100% accurate with Cosine similarity and over 93% results with other similarity measures. Like FaceNet, VGGFace and VGG16 also classify one out of 30 image pairs as the same person where facial hairs or marks are present. The accuracy of the VGG19 model has been constant with all similarity measures where it has only classified one image pair as of the same person with having the same hair cut with a similar-looking face. Figure 5 shows some sample images where models have not classified the siblings and non-siblings as different persons, respectively.

The result analysis shows that VGGFace or VGG16 with Cosine similarity are the best choices for the full-frontal face of siblings. However, if a consistent performance is required with all similarity measures, VGG19 yields the best results. If average accuracy is considered, except FaceNet, all models yield 98% accuracy, suggesting that VGGFace, VGG16, or VGG19 are best when siblings are compared with their full-frontal images.

### 4.3. Experiments on Partial Face

#### 4.3.1. Eyes

Similar experiments have been conducted on the cropped eyes of all pairs of images. A dataset for positive cases and negative cases has been created by cropping the eyes only of all images. All models have been used to compare the images for positive and negative cases using the same similarity measures. Threshold has been deduced for each similarity measure, classifying the same person or different images.

The average accuracy of FaceNet is similar to full-frontal-face comparison. In contrast, its accuracy with separate similarity model has varied from the full-face comparisons, e.g., its accuracy with Euclidean distance has been increased significantly. In contrast, with other similarity measures, minimal variation has been observed. The variation of accuracy might be due to the portion of hair covering the eyes in cropped images.

**Figure 6:**
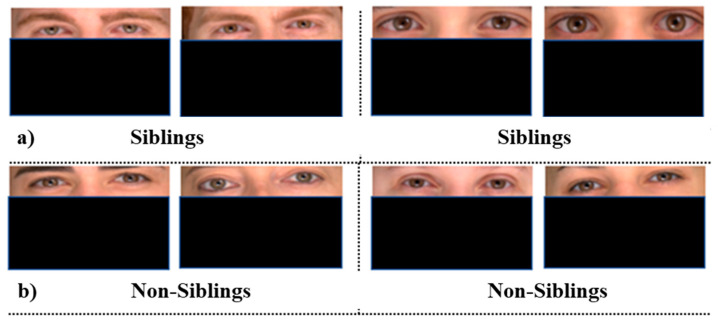
(**a**) Images of the dataset used: two pairs of sibling cropped eyes poses where all four models have classified them as same person and (**b**) images of the dataset used: two pairs of non-sibling cropped eyes poses where all four models have classified them as the same person.

**Figure 7:**
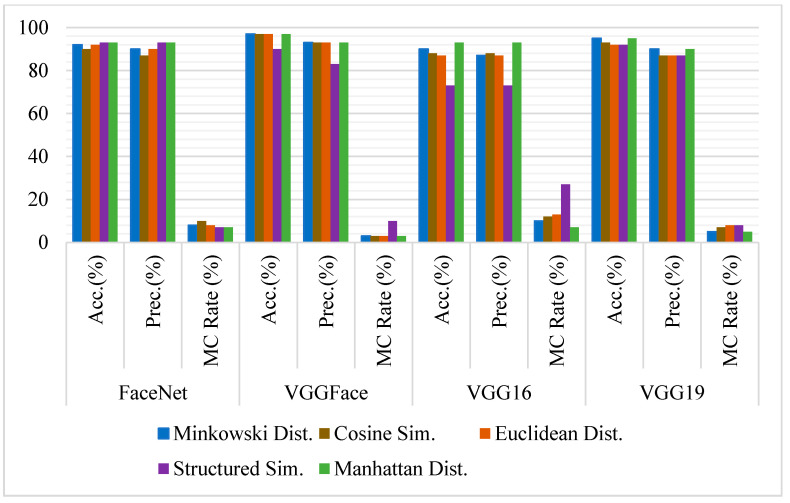
Comparative analysis of FaceNet, VGGFace, VGG16, and VGG19 for face recognition on Cropped eyes image pairs using Accuracy, Precision, and Misclassification rate.

The accuracy of VGG16 has degraded drastically from the previous experiments on the full-frontal-face comparison. On an average, it has yielded a 97% accuracy rate for full-frontal-face comparison. Its accuracy is degraded to 85% on eyes comparison. Its accuracy with each similarity model has also degraded poorly. Some sample image pairs where models have classified siblings and non-siblings as the same person are shown in Figure 6. The accuracy of VGGFace and VGG19 is quite similar to full-frontal-face comparison if an average is calculated. However, the accuracy of these models has varied individually, e.g., the accuracy of VGGFace with structured similarity is degraded to 83% from 97% when full-frontal-face is compared. Similarly, VGG19 is degraded to 92% with cosine compared to 98% throughout the similarity measures while comparing full-frontal-face. Figure 7 contains the graphical comparison of accuracy, precision, and misclassification rates of all four models with each similarity measure.

Though VGG19 and FaceNet have given better results on eyes comparison, it has been observed that VGGFace is the most accurate model with either cosine distance, Euclidean distance, Manhattan, or Minkowski distance similarity, which is 97% when eyes are compared and can be used quite effectively to differentiate between siblings based on eyes comparison only.

#### 4.3.2. Nose

**Figure 8:**
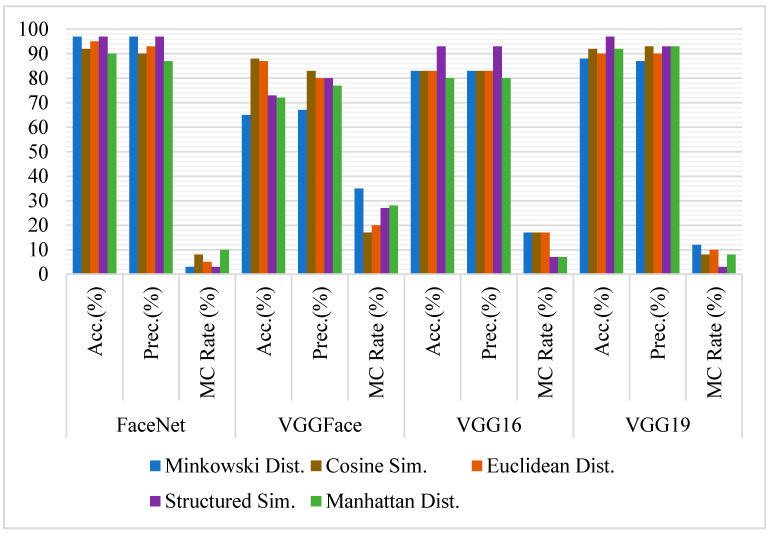
Comparative analysis of FaceNet, VGGFace, VGG16, and VGG19 for face recognition on Cropped nose image pairs using Accuracy, Precision, and Misclassification rate.

In these experiments, the nose of full-frontal-face image pairs for siblings and non-siblings’ subsets taken have been cropped to create new datasets of nose for positive results and negative results. All cropped images have been passed to state-of-the-art models using the same experiments as above, setting thresholds for each similarity measure to evaluate their accuracy. Figure 8 shows the accuracies, precision, and misclassification rates of all four models used.

Unlike full-frontal-face and eyes comparisons, the accuracy of VGGFace has degraded drastically in the case of the nose with all the similarity measures, e.g., its accuracy with Minkowski distance is 65%, whereas it is 97% and 98% with eyes and full-frontal-face, respectively.

The accuracy of VGG16 also has been degraded in case of nose comparison with all similarity measures except with SSIM, where it has yielded the most accuracy of 93%, wherewith all other measures, it has been degraded with at least 7% in comparison to full-frontal-face comparison. Though its accuracy with nose comparison with Manhattan distance is more than the eyes comparison, the average accuracy of VGG16 is the same as eyes comparison.

VGG19 has yielded an accuracy of 97% with Manhattan distance, whereas it is giving 88% with Minkowski distance. Its accuracy with eyes and full-face comparison is constant with all similarity measures, but it is observed that it has varied slightly in the case of the nose.

FaceNet has given the best accuracy of 97% with Minkowski distance, suggesting that it is the best combination for comparing the nose in siblings or similar-looking faces. However, if VGGFace is used with any similarity measures experimented with, the classification rate can be dropped to 65%.

**Figure 9:**
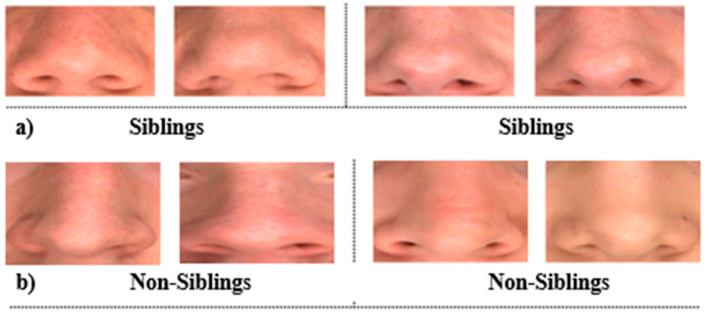
(**a**) Images of the dataset used: two pairs of sibling cropped nose poses where all four models have classified them as the same person. (**b**) Images of the dataset used: two pairs of non-sibling cropped nose poses where all four models have classified them as the same person.

Figure 9 shows some sample pairs of images from sibling and non-siblings cropped nose datasets, respectively, where models have classified them as the same person. It is observed that FaceNet with Minkowski distance and VGG19 with SSIM are the best and effective combinations to differentiate the siblings based on their nose only with 97% accuracy and 3% misclassification rate. In contrast, VGGFace is ineffective in discriminating siblings on their nose only though it has worked perfectly on the full-frontal-face comparison. On the other hand, if the average of all similarity measures is observed, though the results of FaceNet and VGG19 are quite similar (95% and 92%, respectively), FaceNet is producing the best accuracy and precision rate, which is 3% more than VGG19 in the classification of the siblings based on their nose.

#### 4.3.3. Forehead

**Figure 10:**
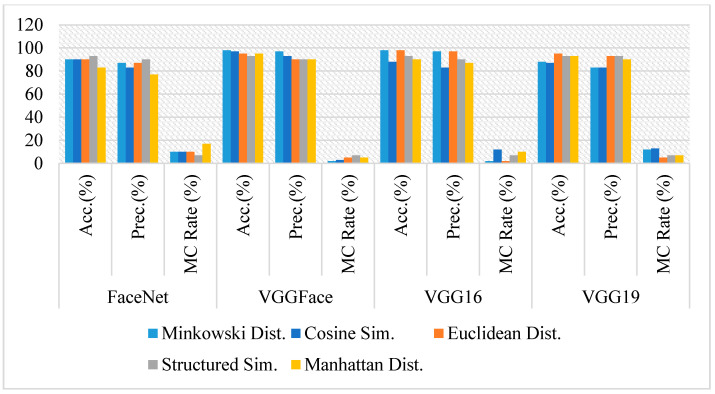
Comparative analysis of FaceNet, VGGFace, VGG16, and VGG19 for face recognition on Cropped forehead image pairs using Accuracy, Precision, and Misclassification rate.

For the experiments on cropped foreheads of the siblings datasets, similar to experiments on eyes and nose datasets, foreheads are cropped from the image pairs of the siblings’ and non-siblings’ datasets created from the HQf dataset of SiblingsDB. All cropped forehead image pairs have been experimented with using all four state-of-the-art models and similarity measures used in previous experiments. As shown in Figure 10, the accuracy, precision, and misclassification rates are evaluated for each model using the image pairs of foreheads of siblings. The accuracy of the FaceNet model while classifying the foreheads of siblings has degraded on an average due to its least accuracy with Manhattan distance. With other similarity measures, its accuracy is at least 90%, similar to the previous experiments. VGGFace model, which has not worked best with the cropped nose, has improved its accuracy massively in foreheads, which is at least 93%.

**Figure 11:**
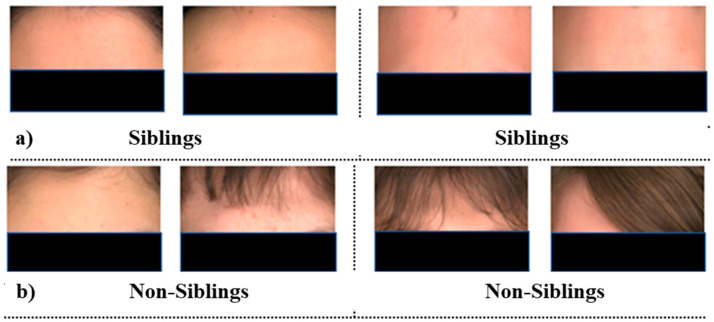
(**a**) Images of the dataset used: two pairs of siblings cropped forehead poses where all four models have classified them as the same person. (**b**) Images of the dataset used: two pairs of non-sibling cropped forehead poses where all four models have classified them as the same person.

Similarly, VGG16 has also increased its performance in the case of the forehead compared to the eyes and nose. Its accuracy varies between 88–98%, with the best accuracy as 98% with Minkowski distance. VGG19′s accuracy also varies from 87% to 95%, which is best with Euclidean distance and worst with cosine similarity. Some sample images of the cropped forehead from sibling and non-sibling pairs are depicted in Figure 11, on which models have classified them as the same person. It is observed that VGGFace or VGG16 with Minkowski distance are the best and most effective combination (98%) to differentiate between siblings based on their foreheads. At the same time, these models have not worked best previously on nose and eyes.

## 5. Discussion

From the results of experiments conducted on full-frontal faces, eyes, nose, and forehead using confusion matrices, Table 1 contains the average accuracy for all four models. It is observed that FaceNet has achieved the least accuracy on full-frontal face comparison for siblings because it has classified more images as of the same person with most of the similarity metrics, whereas VGGFace, VGG16, and VGG19 have achieved similar accuracy. For cropped eyes comparison, VGGFace has given the best accuracy followed by VGG19 and FaceNet, whereas VGG16 achieves the least accuracy due to its classification of images as same based on the similarity metric score and its comparison with a threshold. Similarly, if only nose images are compared, FaceNet has achieved an accuracy of 95%, which is the highest, followed by VGG19, and VGGFace produces the lowest accuracy. FaceNet has yielded the best result for the nose. The similarity metrics for Facenet on nose comparison have classified most of the images as different persons with the least misclassification rate. All models are over 90% accurate on forehead comparison, but VGGFace has produced the highest accuracy of 97%.

Similar to the accuracy metric, the precision metric for all four models is shown in Table 2, which shows that for full-frontal faces, all models are precise between 93–97%, i.e., they are correct when they are classifying the images as of different persons, but FaceNet is the least precise model for full-frontal face comparison. On the other hand, FaceNet and VGGFace give the same and highest precision for eyes comparison, whereas VGG16 is the least precise. On nose comparison, FaceNet has given the precision rate of 93%, which is the highest and in line with its accuracy whereas, VGGFace providing the lowest precision. VGGFace has been the most precise for forehead comparison, and FaceNet has been the least accurate model about the forehead. The highest accuracy for sibling identification for each facial part is shown in bold. 

Table 3 illustrates the misclassification rate metric of all four models while comparing full-frontal-face, eyes, nose, and forehead. It is observed that VGGFace, VGG16, VGG19 have been the most intelligent classifiers when full-frontal-face pose of siblings have been compared due to their least misclassification rate and high accuracy and precision rate as per Table 1 and Table 2. In contrast, FaceNet has given the most misclassification rate for full-frontal-face comparison, i.e., it has wrongly classified the images as of the same person more often than other models.

When the siblings’ eyes are compared, VGGFace has the least misclassification rate, whereas VGG16 has given the highest misclassification rate. For nose comparison, FaceNet has been the most intelligent model where it has classified the least image pairs as same persons and least misclassification rate, and other models have misclassified the image pairs more often.

VGGFace, which was best on eyes and full-frontal-face has given the highest misclassification rate, has misclassified the least image pairs in forehead comparison, whereas FaceNet has misclassified the most image pairs giving the highest misclassification rate.

The experimental results of all four models to differentiate between siblings’ full-frontal-face pose, cropped nose, eyes, and forehead might raise a question of choosing the best FR model among FaceNet, VGGFace, VGG16, and VGG19 in order to discriminate between siblings with either full-face pose or partially available face area.

The comparative analysis above shows that if full-frontal-face is compared, VGGFace, VGG16, or VGG19 can be used, yielding the highest results. Similarly, if only eyes are compared, VGGFace is recommended, giving the highest accuracy even though the precision rate for VGGFace and FaceNet is similar. In comparison between nose areas, FaceNet is the choice of the model due to its highest accuracy and precision rate. VGG16 has produced the highest metrics in forehead comparison to being the choice for forehead comparison. Interestingly, if averages of all metrics are observed, the performance of VGG19 is best even though its accuracy, precision, or misclassification rate is less individually on the areas compared.

## 6. Conclusions

In this paper, a novel framework is presented to explore the performance of four state-of-the-art face recognition models to discriminate siblings using their full-frontal-face, cropped eyes, nose, and forehead with five different similarity measures to classify the sibling facial image pairs.

The results have shown that facial recognition models are robust in distinguishing sibling pairs when full-frontal-face are provided, but the accuracy of models varies when cropped face areas are compared. Experimental results measured the performance when full-frontal faces are provided as well as when cropped face areas (e.g., eyes, nose, and forehead) are provided. The results have shown that if one face recognition model has given the highest accuracy for full-frontal-face, the model will not necessarily classify accurately when part of the face is provided. The converse is also true. Although each model provides different results, on average, their results on each similarity measure also vary on each face area. For example, FaceNet’s accuracy on average is 90%, but its accuracy with Euclidean distance is the least, which has caused its average accuracy to decrease while its accuracy with other similarity measures has been 93%. In contrast, VGGFace has surpassed other models when cropped eyes and foreheads are compared, whereas its accuracy with nose has significantly degraded.

The experiments in this work are performed on the dataset with high-quality images of sibling pairs shot in a controlled environment different from real-world situations. Hence this work might be preliminary but could be used as a basis for future studies in the field of face recognition to determine which state-of-the-art face recognition models can be used in different scenarios to discriminate between siblings or faces that are look-alikes.

There could be multiple further steps to this study. It will be useful to extend this work to assess the performance of state-of-the-art models in real-world situations such as images from CCTVs. Furthermore, a larger sibling database would also be important to evaluate the performance of state-of-the-art models. Another interesting area of improvement could be to combine the best-performing models to create an aggregate framework to discriminate between siblings. A further step to this study could also be to evaluate the accuracy of state-of-the-art models on other parts of the face, e.g., chin, lips, or with a different face pose, e.g., profile pictures of siblings.

## Figures and Tables

**Table 1:** Comparative analysis of FR models using Accuracy Metric.

Face	FaceNet [25]	VGGFace [26]	VGG16 [28]	VGG19 [28]
Full frontal face	90	**98**	**98**	**98**
Eyes	92	**95**	85	93
Nose	**95**	77	83	92
Forehead	88	**97**	93	92
Average	91	92	90	**94**

**Table 2:** Comparative analysis of FR models using Precision Metric.

Face	FaceNet [25]	VGGFace [26]	VGG16 [28]	VGG19 [28]
Full frontal face	93	**97**	**97**	**97**
Eyes	**90**	**90**	83	87
Nose	**93**	77	83	90
Forehead	83	**93**	90	90
Average	90	89	88	**91**

**Table 3:** Comparative analysis of FR models using Misclassification rate Metric.

Face	FaceNet [25]	VGGFace [26]	VGG16 [28]	VGG19 [28]
Full frontal face	10	**2**	**2**	**2**
Eyes	8	**5**	15	7
Nose	**5**	23	17	8
Forehead	12	**3**	7	8
Average	9	8	10	**6**

## Data Availability

Not Applicable.
